# Устранение дефицита йода – забота о здоровье нации. Экскурс в историю, научные аспекты и современное состояние правового регулирования проблемы в России

**DOI:** 10.14341/probl13154

**Published:** 2022-07-27

**Authors:** Е. А. Трошина

**Affiliations:** Национальный медицинский исследовательский центр эндокринологии

**Keywords:** щитовидная железа, йод, Российская Федерация, профилактика

## Abstract

В статье представлены актуальные данные о распространенности заболеваний щитовидной железы (ЩЖ), связанных с дефицитом йода, в России, сделаны акценты на особенностях коморбидности йододефицитных и аутоиммунных патологий ЩЖ, методах оценки йодной обеспеченности населения. Приводятся сведения об изучении и профилактике йододефицитных заболеваний (ЙДЗ) в СССР, Российской Федерации. Подробно иллюстрируется история законодательных инициатив, направленных на устранение дефицита йода в питании и предотвращение ЙДЗ. Предлагаются пути решения проблемы йододефицита на современном этапе как на федеральном, так и на региональном уровнях.

## Abstract (en)

The article presents current data on the prevalence of thyroid diseases associated with iodine deficiency in Russia, focuses on the features of the comorbidity of iodine deficiency and autoimmune thyroid pathologies, methods for assessing the iodine supply of the population. Information about the study and prevention of iodine deficiency diseases (IDD) in the USSR and the Russian Federation is given. The history of legislative initiatives aimed at eliminating dietary iodine deficiency and preventing IDD is illustrated in detail. The ways of solving the problem of iodine deficiency at the present stage, both at the federal and regional levels, are proposed.

Йододефицитные заболевания (ЙДЗ) — это все нарушения здоровья, развитие которых связано с хроническим дефицитом йода в питании и полностью предотвратимые при его устранении. По данным ВОЗ, заболевания щитовидной железы (ЩЖ) среди эндокринных нарушений занимают второе место после сахарного диабета, причем до 80% из них вызваны хроническим дефицитом йода в питании [1]. Данные многих исследований убедительно свидетельствуют в пользу высокой распространенности коморбидности йододефицитных и аутоиммунных заболеваний ЩЖ у лиц, проживающих в условиях некомпенсированного дефицита йода, при этом многие фундаментальные механизмы их патогенетических взаимовлияний нуждаются в изучении в первую очередь для научного обоснования, создания и внедрения новых диагностических технологий, в т.ч. с использованием возможностей искусственного интеллекта и самообучающихся нейросетей. Сегодня доказано, что аутоиммунные и ЙДЗ ЩЖ являются мультифакторными патологиями, не исключено, что генетическая предрасположенность к их развитию и взаимосочетанию представлена определенным полиморфизмом генов регуляции иммунного ответа, однако главным триггерным фактором, приводящим к манифестации заболеваний ЩЖ, служат факторы окружающей среды, наиболее изученным из которых является хронический дефицит йода в питании [2][3].

Результаты масштабных исследований, проведенных за последние 20 лет в ФГБУ «НМИЦ эндокринологии» по поручению Минздрава России, убедительно доказали, что потребление йода россиянами составляет не более 80 мкг в сутки, что в 2–3 раза меньше рекомендованной нормы 150–250 мкг в сутки [4]. Официальная статистика заболеваемости отмечает не только лидирующие позиции заболеваний ЩЖ в структуре всей эндокринной патологии (27,6%, Росстат, 2020 г.), ведущее место суммарной доли зоба и синдрома врожденной йодной недостаточности среди всей патологии ЩЖ, но и динамику роста заболеваемости патологиями ЩЖ с увеличением вдвое за последние 10 лет [5]. Очевидно, что это связано с хроническим дефицитом йода в питании. Причем данные исследований показали, что фактическая распространенность зоба в 10 раз превышает регистрируемую, а наиболее уязвимыми в плане развития угрожающих последствий йодного дефицита являются беременные, кормящие женщины и дети [6][7]. ЙДЗ ассоциированы и с весомыми финансовыми затратами на проведение диагностических и лечебных мероприятий, хирургических вмешательств, терапии радиоактивным йодом, реабилитации, постоянного динамического наблюдения, биологического эпидемиологического мониторинга.

Нужно подчеркнуть, что большая часть йода, поступающего с пищей, выводится почками, именно поэтому экскреция йода с мочой служит хорошим маркером потребления йода. В течение суток уровень йода в моче может варьировать у одного и того же человека. Определение экскреции йода с мочой используют для популяционных исследований и оценки обеспеченности йодом региона. Популяционные исследования этого показателя у населения сглаживают его вариативность. В связи с широким разбросом индивидуальных показателей итоги эпидемиологических исследований рекомендуется оценивать не по среднему арифметическому, а по медиане. В настоящее время медиану уровня йода в моче рассматривают как основной эпидемиологический признак, характеризующий йодную обеспеченность того или иного региона. Этот показатель быстро реагирует на изменения в потреблении йода и потому имеет важнейшее значение не только для оценки эпидемиологической ситуации, но и для контроля эффективности профилактических мер по предотвращению йододефицитных заболеваний [8].

Важно отметить, что ВОЗ еще в 1994 г. включила уровень неонатального тиреотропного гормона выше 5 мМЕ/л в индикаторы оценки степени тяжести йодного дефицита [9], однако до сих пор не совсем понятно, каким образом рассчитывались точки отсечения показателя неонатальной гипертиреотропинемии как индикатора тяжести дефицита йода. При этом бесспорно, что частота неонатальной гипертиреотропинемии в качестве, например, индикатора мониторинга эффективности профилактических программ может иметь ряд преимуществ, поскольку скрининг на врожденный гипотиреоз охватывает всех новорожденных, а анализ результатов не требует дополнительного финансирования. В России исследовательская работа, направленная на уточнение этого важного вопроса, проводится учеными под руководством профессора Л.А. Суплотовой, и ее результаты будут опубликованы в ближайшем будущем.

Не лишним будет сделать акцент и на том факте, что «полиморфизм» методик и биосред для определения уровня йода, предлагаемый сегодня коммерческими лабораториями, нередко вводит в заблуждение как пациентов, так врачей, а получаемые результаты невозможно интерпретировать с точки зрения доказательности. Не вдаваясь в подробности оценки присутствующих на «лабораторном рынке» подходов к оценке «содержания йода», подчеркнем, что аналитическое определение йода до сегодняшнего дня, несмотря на наличие многих методов, остается весьма сложным и трудоемким. Это связано с его летучестью, возможностью вступать в окислительно-восстановительные реакции с компонентами анализируемого вещества, поливалентностью и в ряде случаев с малой концентрацией [10]. Именно поэтому определение йода в волосах, ногтях, слюне и т.д. не имеет доказательной базы.

Медианная концентрация йода в моче позволяет судить о йодной обеспеченности в ходе эпидемиологических исследований. Низкий уровень йодной обеспеченности неизбежно приводит к развитию ЙДЗ, в т.ч. тиреопатий и поражений мозга различной степени выраженности. Сегодня очевидно, что какой бы гениальной наследственной информацией, полученной от родителей, не обладал ребенок, при недостатке гормонов ЩЖ в условиях дефицита йода она не будет реализована — произойдет необратимое нарушение психического развития. Особенно важное значение имеет достаточный уровень гормонов ЩЖ для формирования нервной системы человека в эмбриональном периоде, следовательно, будущий интеллект ребенка напрямую зависит от йодной обеспеченности матери во время беременности.

К ЙДЗ ЩЖ относятся диффузный нетоксический зоб, узловой и многоузловой коллоидный нетоксический и токсический зоб (с развитием функциональной автономии и тиреотоксикозом), гипотиреоз (в случае тяжелого дефицита йода) [11][12]. Для всех возрастных групп населения в условиях дефицита йода на порядки возрастает риск развития патологии ЩЖ, в т.ч. с формированием узловых образований, нарушениями ее функции, формируя опасность усугубления сердечно-сосудистых патологий у людей старших возрастных групп. В пожилом возрасте последствия длительного (пожизненного) дефицита йода лежат в основе прямых рисков сердечно-сосудистых заболеваний, в первую очередь нарушений ритма сердца [13–15].

В России наблюдается тенденция к росту заболеваемости узловым и многоузловым зобом у лиц старше 50 лет. За последние пять лет, по данным Росстата, обращает на себя внимание рост узловых форм зоба у пожилых людей с 70,5 до 103,6 случая на 100 тысяч населения. Средняя заболеваемость в этой группе была 88,5 на 100 000 населения, средний ежегодный прирост заболеваемости составил 4%. Также наибольшая заболеваемость тиреотоксикозом установлена у лиц старше 60 лет. Число новых случаев увеличилось с 8,5 до 15,7 на 100 000 населения, ежегодный прирост заболеваемости составил 3,4%. За последние 10 лет отмечен рост распространенности и аутоиммунных патологий ЩЖ (АИЗ ЩЖ), и ассоциированных с ними дисфункций: тиреоидита (медиана ежегодного прироста распространенности — 15,9 случая на 100 000 человек); гипотиреоза (медиана ежегодного прироста распространенности — 24,9 случая на 100 000 человек); тиреотоксикоза (медиана ежегодного прироста распространенности — 3,1 случая на 100 000 человек) [5].

Почему эти тенденции очень опасны? Наиболее важным органом-мишенью при функциональных нарушениях ЩЖ является сердце. Тиреотоксикоз может приводить к тахикардии, наджелудочковым аритмиям, ухудшению течения стенокардии, застойной сердечной недостаточности, а также обострению ранее существовавших заболеваний сердечно-сосудистой системы [16]. У больных тиреотоксикозом и фибрилляцией предсердий повышен риск тpoмбoэмбoлий, и в некоторых случаях такие больные впервые поступают в клинику с явлениями инсульта и других эмболических осложнений. Кроме того, при тиреотоксикозе отмечается склонность к гиперкоагуляции.

Многолетний опыт использования йодированной соли в регионах с йодной недостаточностью убедительно показал, что длительная профилактика в конце концов снижает частоту тиреотоксикоза в популяции прежде всего за счет устранения факторов, приводящих к формированию многоузловых токсических зобов. Сегодня накапливаются данные и о том, что восполнение дефицита йода влияет на развитие АИЗ ЩЖ. Так, показано, что йод влияет на активность НАДФ-оксидазы, подавляя ее активность, оказывая влияние не только на циклический аденозинмонофосфат, но и на более дистальные цепи. При этом йод не снижает экспрессию мРНК DUOX2 (изоформа двойной оксидазы, принадлежащей к семейству НАДФ-оксидаз (NOX), которая экспрессируется без стимулирующего влияния тиреотропного гормона, но уменьшает образование высокогликозилированной (активной формы) фермента). Не исключено, что этот эффект защищает клетки ЩЖ от окислительного стресса в результате избыточной экспрессии и гиперактивности Н2О2 генерирующей системы [17].

Таким образом, профилактика дефицита йода — это, по сути, профилактика практически всей патологии ЩЖ и ее осложнений. Важно, что ЙДЗ можно полностью предотвратить. Понимание этого формировалось на основе многих и многих исследований и наблюдений. Так, изучение эндемического зоба в России началось в конце ΧІΧ в., когда во многих губерниях тогдашней России были описаны многочисленные случаи эндемического зоба (ЭЗ). Самые ранние исследования ЭЗ в России проведены Николаем Кашиным в Восточной Сибири и Николаем Лежневым в Пермской губернии. Первая книга об ЭЗ была написана именно Лежневым, в ней он констатировал: «Зоб имеет важное национальное значение, а борьба с ним является жизненно необходимой». Эти слова актуальны и по сей день.

В СССР стартом системной работы по изучению и профилактике заболеваний, связанных с дефицитом йода, можно назвать приказ №382 1929 г. Народного комиссара здравоохранения Н.А. Семашко, выпущенный во исполнение постановления Совета Народных комиссаров РСФСР от 4 января 1929 г. о проведении работы по обследованию и изучению эндемии зоба в Марийской автономной области (к этому времени были получены первые данные о высокой распространенности зоба на территории области). На тот момент стало понятным, что работа по профилактике зоба должна вестись содружественно специалистами эндокринологами и санитарными врачами. Первые экспедиции в регионы объединяли представителей двух служб — эндокринологической и санитарно-гигиенической. 1929 г. можно считать началом планомерной работы по изучению ЙДЗ и организации их профилактики.

Нужно отметить, что все инициированные в то время меры имели исключительно важное значение, а именно: в СССР была обозначена проблема дефицита йода и заболеваний, вызванных этим дефицитом (прежде всего — заболеваний ЩЖ, кретинизма, репродуктивных потерь); принят ряд организационных решений, направленных на устранение дефицита йода и профилактику ЙДЗ; было положено начало созданию сети противозобных центров, стартовали научные исследования, посвященные различным аспектам этиологии и патогенеза зоба; шло активное накопление знаний о проблеме в целом, велись обследования населения в проблемных регионах страны, закладывались основы массовой и индивидуальной йодной профилактики. Все вышеизложенное стало основой для принятия дальнейших важнейших решений, направленных на йодную профилактику в СССР. Еще одними важными моментами, которые были обозначены в начале прошлого века, стали обоснование необходимости и начало внедрения индивидуальной йодной профилактики, прежде всего у беременных женщин и детей.

Первые научные исследования ЭЗ в СССР того времени, безусловно, связаны с именем О.В. Николаева — выдающегося хирурга-эндокринолога и специалиста по профилактике ЭЗ. В одной из своих первых публикаций «Этиология эндемического зоба», вышедшей в 1932 г., он определил важность проблемы ЭЗ и предложил обеспечение йодированной солью населения, проживающего на подверженных этому заболеванию территориях, назвав йодированную соль «полноценной солью», содержащей жизненно важный микроэлемент — йод.

Работы О.В. Николаева и его коллег, важнейшие данные, полученные при обследовании населения различных регионов СССР, и стали основанием для издания в 1956 г. приказа № 37-М Министерства здравоохранения СССР «Об улучшении работы по борьбе с эндемическим зобом». Этот приказ определил стратегию профилактики ЭЗ в стране, ориентированную на использование йодированной соли в питании, мониторинг состояния здоровья населения специалистами противозобных диспансеров. Более того, в 1960-е гг. в СССР было проведено геохимическое обследование всей территории страны и создана карта «биогеохимических провинций» с низким содержанием йода в почве и воде. В этих районах был установлен более жесткий контроль над обеспечением населения йодированной солью, а в период между 1965 и 1969 гг. в СССР было проведено два всесоюзных обследования распространенности ЭЗ. Эти обследования проводились в основном специальными экспедиционными группами, состоящими из медицинских работников противозобных диспансеров. К этому времени в стране было создано 63 таких диспансера, они располагались во всех «эндемичных по зобу» регионах.

В результате масштабных исследований было установлено, что распространенность ЭЗ существенно снизилась, что было связано с совершенствованием системы профилактики ЭЗ, в первую очередь увеличением производства и поставок йодированной соли. В результате в начале 1970-х гг. было официально объявлено о практически полном устранении ЭЗ в СССР. В то же время мониторинг ЭЗ как на всесоюзном, так и на региональном уровне стал постепенно ослабевать, общегосударственный контроль над ситуацией был в значительной мере утрачен, а заболеваемость ЭЗ более не отслеживалась [18].

Контрольно-эпидемиологические исследования ЙДЗ и анализ их результатов в России были инициированы Министерством здравоохранения России лишь в 1999 г., после выхода соответствующего Постановления Правительства Российской Федерации, подписанного В.В. Путиным, проводились и проводятся по поручению Министерства здравоохранения под руководством главного внештатного специалиста эндокринолога России, директора, а ныне президента ФГБУ «НМИЦ эндокринологии» Минздрава России академика И.И. Дедова, и позволили создать за последние 20 лет карту йодной обеспеченности населения страны, а также научно обосновать ­необходимость ­законодательного закрепления системной йодной профилактики йодированной солью. За этот период вышла и целая серия постановлений Главного государственного санитарного врача, направленных на усиление профилактики дефицита йода в питании россиян.

Было обследовано более 100 000 человек в различных регионах России, подтвержден факт йодного дефицита на всей территории страны. Выделены группы высокого риска развития ЙДЗ — беременные, кормящие женщины и дети. Доказано, что модель «добровольного» использования йодирования соли не дала ожидаемых результатов в плане удовлетворения оптимальной потребности населения в йоде и снижения заболеваемости, в т.ч. в группах риска. Установлено, что в Российской Федерации йодированную соль в питании употребляет менее 30% населения, несмотря на то, что повсеместное использование йодированной соли является наиболее простым, эффективным и безопасным способом коррекции йодного дефицита. Аналитические результаты исследований и комплексная оценка данных Росстата регулярно публикуются и предоставляются в Минздрав России [4][19].

Принимая во внимание все вышеизложенное, очевидно, что вопрос ликвидации йододефицита должен рассматриваться в контексте государственных инициатив, направленных на создание условий для ведения здорового образа жизни в любом возрасте. Для успешного решения вопроса профилактики ЙДЗ необходим соответствующий федеральный закон (ФЗ). Проект такого ФЗ и был разработан Минздравом России. Почему же до сих пор проект не стал законом? Тем более что в основе государственных стратегий многих стран мирового сообщества, направленных на ликвидацию ЙДЗ, лежит именно принятие закона, предусматривающего использование йодированной соли в качестве средства массовой (популяционной) йодной профилактики. Беспрецедентный опыт стран, устойчиво устранивших дефицит йода, убедительно свидетельствует, что использование йодированной соли для популяционной профилактики ЙДЗ является универсальным, безопасным, эффективным и экономически обоснованным. При этом важно отметить, что:

Действительно, использование популяционной йодной профилактики при помощи йодирования соли позволило странам, ранее входившим в состав СССР, достичь успеха в укреплении здоровья своих граждан, полностью ликвидировав йодный дефицит или существенно снизив его тяжесть. Тем не менее на сегодняшний день лишь Россия и Украина не имеют законодательного регулирования профилактики дефицита йода [20][21]. Так почему же законопроект до сих пор не стал законом в России?

Справка: Законодательное регулирование профилактики ЙДЗ

Как уже сказано выше, в России на протяжении последних 20 лет предпринимаются попытки осуществления законодательного регулирования йодной профилактики. Ряд законопроектов разрабатывался Минздравом России, однако на этапах оценки регулирующего воздействия (ОРВ) не находил поддержки.

Как же развивались события? В принятой в начале 1990-х гг. исторической резолюции Всемирной ­Ассамблеи Здравоохранения (ВОЗ) нашла отражение чрезвычайно высокая медико-социальная значимость ­недостаточности йода в питании населения земного шара. Озабоченность медиков вопросами обеспечения человека достаточным количеством йода на протяжении всей жизни вызвана тем, что йод относится к тем микроэлементам, которые не обладают способностью накапливаться в организме и потому должны постоянно пополняться с пищей.

В 1990 г. на Всемирной встрече на высшем уровне Россия подписала Конвенцию о правах ребенка, взяла на себя обязательство устранить ЙДЗ. В 1999 г. Правительством РФ принято Постановление N1119 «О мерах по профилактике заболеваний, связанных с дефицитом йода», однако всеобщего йодирования соли в нем не предусматривалось. Спустя 6 лет, на Сессии Генеральной Ассамблеи ООН, Комитет по правам ребенка рассмотрел отчеты стран по выполнению обязательств Конвенции, выразил обеспокоенность по поводу расстройств, связанных с сохраняющейся недостаточностью йода в России, и призвал принять закон о повсеместном йодировании соли. За период 1990-2022 гг. в 126 из 130 стран мира, где существовал дефицит йода, принято законодательство по йодированию соли.

Работа над законом о профилактике ЙДЗ инициирована Минздравом России с 2003 г. Однако на протяжении многих лет его принятие стойко блокируется ведомствами экономического блока. Считаем правильным привести ниже «историю законопроекта» и причины его непринятия:

В 2016 г. Минздравом вновь был подготовлен проект соответствующего ФЗ по йодированию соли, он претерпел коррекцию в связи с неприятием идей всеобщего йодирования соли и стал более лояльным, а именно предполагалось, что «Пищевая поваренная соль выварочная, каменная, садочная и самосадочная сортов “Экстра” и высший, помолов № 0 и № 1, производимая на территории Российской Федерации и импортируемая в Российскую Федерацию, должна быть обогащена йодатом калия», «Пищевая поваренная соль, используемая при промышленном изготовлении пищевых продуктов и при приготовлении пищевых продуктов в организациях общественного питания, должна быть обогащена йодатом калия в концентрациях, устанавливаемых уполномоченным федеральным органом исполнительной власти». Данный проект ФЗ опять не прошел согласования с такими министерствами, как Минпромторг, Минэкономразвития, Минсельхоз и ФАС. В результате была подготовлена «усеченная» версия законопроекта, согласно которому йодированной должна быть только соль, используемая для досаливания (в солонке) в организациях общественного питания, а также в хлебе, используемом при организации общественного питания. Эта версия законопроекта смогла бы защитить здоровье лишь незначительной части граждан, однако и она получила негативные отзывы ведомств экономического блока.

Самый последний проект ФЗ «О профилактике заболеваний, вызванных дефицитом йода» был разработан Министерством здравоохранения России во исполнение пункта 50 плана мероприятий по реализации Стратегии повышения качества пищевой продукции в РФ до 2030 г., утвержденной распоряжением Правительства РФ от 19 апреля 2017 г. № 738-р, подпункта «а» пункта 1 перечня поручений Президента РФ В.В. Путина от 3 июля 2018 г. № Пр-1136, поручения Заместителя председателя Правительства РФ Т.А. Голиковой от 15 июня 2018 г. № ТТ-П12-3408.

17 апреля 2020 г. Минздравом России проект федерального закона представлен в Правительство Российской Федерации [22].

Проект федерального закона согласован Минобрнауки России, Минпромторгом России, Роспотребнадзором, Министерством просвещения Российской Федерации, Министерством культуры Российской Федерации, Министерством обороны Российской Федерации, Федеральной службой исполнения наказаний. С Минэкономразвития России и Минсельхозом России оформлены таблицы разногласий.

В Институт законодательства и сравнительного правоведения при Правительстве Российской Федерации, который концептуально поддержал законопроект, но имел ряд замечаний, была направлена таблица с позицией Минздрава России по представленным замечаниям.

Проект федерального закона одобрен 25 мая 2020 г. на заочном заседании рабочей группы в полном составе по реализации механизма «регуляторной гильотины» в сфере здравоохранения при подкомиссии по совершенствованию контрольно-надзорных и разрешительных функций федеральных органов исполнительной власти при Правительственной комиссии по проведению административной реформы. Получено заключение Минюста России на проект федерального закона. На проект федерального закона получены положительные заключения ФГБУ «НМИЦ эндокринологии» Минздрава России и ФГБУ «ЦНИИОИЗ» Минздрава России. Кроме того, ФГБУН «ФИЦ питания и биотехнологии» представлены данные по особенностям использования йодированной соли. По итогам обсуждения проекта федерального закона на площадке Аналитического центра при Правительстве РФ в рамках совместных заседаний членов рабочих групп от экспертного и делового сообщества пореализации механизма «регуляторной гильотины» в сфере здравоохранения, санитарно-эпидемиологического благополучия, оценки соответствия (технического регулирования) проект федерального закона доработан, а именно проведены следующие мероприятия.

Законопроект был поддержан многими федеральными органами исполнительной власти, но при рассмотрении с точки зрения оценки регулирующего воздействия (ОРВ) Минэкономразвития вновь не получил поддержки. Основная аргументация при ОРВ — законопроект «содержит положения, вводящие избыточные обязанности, запреты и ограничения для физических и юридических лиц в сфере предпринимательской и иной экономической деятельности или способствующие их введению» (№ 10890-АХ/Д26и от 09.04.2021, Минэкономразвития, «Заключение об ОРВ на проект ФЗ “О профилактике заболеваний, вызванных дефицитом йода”»). Масса негативных неофициальных отзывов на проект ФЗ поступила со стороны производителей пищевых и биологически активных добавок с йодом. Минздрав после доработки (формирование ответов на замечания, составление таблицы разногласий) вновь, согласно процедуре, направил законопроект в Минэкономразвития на отзыв (оценка регулирующего воздействия).

Складывается впечатление, что, несмотря на высокую распространенность и заболеваемость ЙДЗ, на успешный опыт стран мирового сообщества по популяционной профилактике с использованием йодированной соли, «угроза бизнесу» в России, где люди продолжают испытывать хронический дефицит йода с развитием всего спектра патологий, представляется более серьезной, чем угроза здоровью. Нельзя не сказать о важности принимаемых мер, направленных на профилактику ЙДЗ со стороны Рос­потребнадзора. Так, изменения, внесенные в СанПиН 2.3/2.4.3590-20 «Санитарно-эпидемиологические требования к организации общественного питания населения», позволили обеспечить обязательное использование йодированной солив организованном питании детей. Казалось бы, логично, что объединение усилий двух ключевых ведомств, ответственных за здоровье граждан страны, Мин­здрава и Роспотребнадзора, в доработке и продвижении законопроекта «О профилактике заболеваний, вызванных дефицитом йода», было бы исключительно важным. Тем не менее в 2022 г. Роспотребнадзор разработал новый проект ФЗ «О проведении эксперимента в 2022–2023 гг. в отдельных субъектах РФ (йододефицитных регионах) по внедрению в производство обогащенных йодом пищевых продуктов и использованию их в медицинских, дошкольных, образовательных, санаторно-курортных и иных организациях, а также в оптовой и розничной торговой сети», предусматривающий (в его первой редакции) использование разнообразных пищевых добавок с йодом в качестве средств популяционной профилактики [22].

Сформировано и официально представлено компетентное консолидированное критическое мнение ученых и врачей, входящих в Российскую ассоциацию эндокринологов, по этому проекту ФЗ с указанием, что ни в одной стране мира ни БАДы, ни пищевые добавки с йодом не используются в качестве средств популяционной йодной профилактики вследствие отсутствия доказательств их безопасности и дороговизны.

Нужно отметить, что из 14 субъектов, отнесенных к экспериментальным, 12 не поддержали предложение войти в эксперимент. В результате проект ФЗ претерпел некоторые изменения и изменилось его название. В последней редакции проект ФЗ называется «О проведении эксперимента в 2022–2023 гг. в отдельных субъектах Российской Федерации (йододефицитных регионах) по внедрению в производство обогащенных йодом пищевых продуктов (прежде всего соли) и использованию их в организациях общественного питания, медицинских, образовательных и иных организациях» [22]. В качестве пилотных регионов выбраны «йододефицитные регионы» — Новосибирская и Архангельская области, давшие согласие на участие в эксперименте. В проекте ФЗ указано, что Новосибирская область — территория с «выраженной проблемой йододефицита», Архангельская область — «испытывает существенный дефицит йода». Помимо того, что в оценке тяжести дефицита йода нет дефиниций «выраженный» и «существенный», следует отметить, что эпидемиологические исследования, подтверждающие наличие йодного дефицита и определившие его тяжесть, были проведены в данных регионах очень давно либо не проводились (Архангельская область — 2000 г., Новосибирская область — не проводились). Это имеет принципиальное значение, поскольку без актуальных первичных данных о тяжести йодного дефицита и фактической распространенности ЙДЗ невозможно будет делать выводы об эффективности предполагаемых законопроектом мероприятий.

Недоумение вызывает и утверждение, включенное в проект ФЗ: «Пищевая поваренная соль, обогащенная йодом, не должна являться единственным выходом из сложившейся ситуации, в том числе на фоне рекомендаций о необходимости общего сокращения потребления соли, особенно для лиц с патологией сердечно-сосудистой системы, сахарным диабетом, ожирением и избыточной массой тела». Со всей ответственностью констатируем, что снижение потребления соли в рационе и ее обогащение соединениями йода не противоречат и не мешают друг другу.

Исключительно важно в стремлении устранить дефицит йода в питании россиян всем министерствам и ведомствам действовать согласованно и конструктивно, основываясь на доказанные опытом всего мирового сообщества способы решения проблемы. Оценивая масштаб угрозы некомпенсированного дефицита йода для здоровья и интеллекта нынешних и будущих поколений, очевидно, что закон о популяционной йодной профилактике йодированием соли в России необходим, учитывая тот факт, что принятие такого закона — процесс длительный, выходящий за рамки сугубо медицинских аспектов, требующий комплексных согласований, исключительно важно реализовать возможность инициации и проведения незамедлительной профилактики ЙДЗ на региональном уровне. В этой связи актуальным шагом может стать формирование профилактического процесса, базирующегося на соответствующей нормативно-правовой базе в каждом субъекте РФ — разработка и реализация целевых региональных программ по профилактике ЙДЗ, что полностью гармонизировано со стратегическим курсом Минздрава России. Так, приказом Минздрава России от 15.01.2020 №8 утверждена «Стратегия формирования здорового образа жизни населения, профилактики и контроля неинфекционных заболеваний на период до 2025 года». Для решения задач Стратегии в числе основных направлений указана ликвидация микронутриентной недостаточности, прежде всего дефицита йода.

Что же должно лежать в основе профилактики ЙДЗ на региональном уровне? Опыт реализации программ профилактики имеет практически половина субъектов РФ, наибольшая активность в этой сфере пришлась на период 2000–2005 гг. Анализ результативности программ, проведенный Минздравом, показал, что успешными в части достижения основных индикаторов йодной обеспеченности были только те регионы, которые пошли по пути внедрения массовой йодной профилактики йодированной солью — средством с доказанными эффективностью и безопасностью и наладили стройную систему межведомственного взаимодействия [4]. В качестве одного из примеров межведомственного взаимодействия можно привести изданное в 2016 г. распоряжение Правительства Республики Тыва «Об утверждении межведомственного плана мероприятий по формированию здорового образа жизни у населения Республики Тыва на 2016–2018 годы», согласно которому предприятиям пищевой и перерабатывающей промышленности рекомендовано использовать йодированную соль при производстве молочной продукции и хлебобулочных изделий; управлению Роспотребнадзора и Министерству сельского хозяйства и продовольствия поручено проводить контрольные мероприятия по использованию йодированной соли при производстве продуктов питания [23]. Данные мероприятия существенно повлияли на обеспеченность населения республики йодом.

Исследования, проведенные НМИЦ эндокринологии в этом регионе в 2020 г., подтверждают существенный успех, который реализуется в сбережение здоровья жителей республики. Регион, имеющий тяжелый природный дефицит йода, ныне по распространенности и заболеваемости патологиями ЩЖ сопоставим с российскими регионами легкого йодного дефицита (республика Крым, Брянская область) [24][25].

Исследования, направленные на оценку йодной обеспеченности, а также уточнение патогенетических основ коморбидности йододефицитных и АИЗ ЩЖ, проведены специалистами НМИЦ эндокринологии в Чеченской республике и Тульской области в 2022 г. Полученные данные убедительно свидетельствуют о частом сочетании узловых форм зоба и хронического аутоиммунного тиреоидита, научное объяснение этого факта — впереди, однако уже сегодня есть понимание, что обнаружение подобного рода ассоциаций может стать предметом первой необходимости для обучения нейронных сетей распознавать опухоли среди огромного массива коллоидных узлов на фоне измененной структуры ЩЖ. Такая работа стартовала и продолжается.

Говоря о профилактике ЙДЗ, нельзя не затронуть экономическую составляющую. Сегодня один пациент с верифицированным ЙДЗ «обходится» государству в 10 222 рубля в год (без учета оплаты больничных листов, реабилитации, затрат на содержание инвалидов (кретинизм, вызванный йодным дефицитом), предотвращение репродуктивных потерь, связанных с дефицитом йода, йодную профилактику лекарственными препаратами йодида калия в группах риска ЙДЗ). Очевидно, что снижение заболеваемости ЙДЗ является экономически обоснованным.

Принципиально важно, что для достижения успеха в борьбе с ЙДЗ и значимых результатов по ликвидации дефицита йода в питании населения в масштабах страны необходимо участие каждого ее субъекта, в которых будут проводиться профилактические мероприятия с учетом самых различных особенностей территории в рамках соответствующих региональных программ [6][26]. Разработка и внедрение таких программ, с одной стороны, могут быть определены соответствующими подзаконными актами, которые последуют за ФЗ «О профилактике заболеваний, связанных с дефицитом йода» и станут его практической реализацией на местах, но, с другой стороны, ничто не сдерживает инициацию системной работы по профилактике ЙДЗ на региональном уровне уже сейчас, более того, необходимость такой работы очевидна [27][28].

Как уже было сказано выше, вся история научных исследований, эпидемиологического мониторинга, профилактики и лечения заболеваний, вызванных дефицитом йода, неразрывно связана с эндокринологией и эндокринологами. Сегодня принципиальная консолидированная позиция всех специалистов, объединенных Российской ассоциацией эндокринологов, в отношении необходимости принятия ФЗ о популяционной йодной профилактике ЙДЗ позволила научно обосновать его целесообразность и, по сути, поднять данный вопрос на уровень Президента и Правительства страны. Это исключительно важно, поскольку простое решение, которое нужно в итоге принять на федеральном уровне, несомненно, позволит сохранить здоровье людей и будет влиять на интеллект нынешнего и будущих поколений.

## ДОПОЛНИТЕЛЬНАЯ ИНФОРМАЦИЯ

Источники финансирования. Статья подготовлена в рамках проекта, реализуемого по гранту РНФ «Научное обоснование, разработка и внедрение новых технологий диагностики коморбидных йододефицитных и аутоиммунных заболеваний щитовидной железы, в том числе с использованием возможностей искусственного интеллекта», № 22-15-00135, https://rscf/ru/project/22-15-00135/

Конфликт интересов. Авторы декларируют отсутствие явных и потенциальных конфликтов интересов, связанных с публикацией настоящей статьи.

Участие авторов. Автор одобрил финальную версию статьи перед публикацией, выразил согласие нести ответственность за все аспекты работы, подразумевающую надлежащее изучение и решение вопросов, связанных с точностью или добросовестностью любой части работы
